# Piezo1 in atherosclerosis: a multifaceted mechanosensor bridging mechanotransduction, inflammation, and emerging therapies

**DOI:** 10.3389/fcvm.2026.1979257

**Published:** 2026-10-28

**Authors:** Tianrui Liu, Yongmei Nie

**Affiliations:** 1 School of Clinical Medicine, Southwest Medical University, Luzhou, China; 2 Department of Cardiovascular Surgery, The Affiliated Hospital of Southwest Medical University, Luzhou, China; 3 Key Laboratory of Cardiovascular Remodeling and Dysfunction, Luzhou, China

**Keywords:** atherosclerosis, inflammation, mechanotransduction, pharmacotherapy, Piezo1

## Abstract

The pathogenesis of atherosclerosis is closely associated with dysregulated mechanosensation mediated by the Piezo1 channel. Piezo1 is expressed in vascular endothelial cells, smooth muscle cells, macrophages, and neutrophils, where it transduces hemodynamic shear stress (e.g., oscillatory shear stress, OSS) and extracellular matrix stiffness into pathological signals. At the molecular level, Piezo1 drives endothelial inflammation through the Ca^2+^/calmodulin (CaM)/calcium/calmodulin-dependent protein kinase II (CaMKII)-focal adhesion kinase (FAK)/Src-Yes-associated protein (YAP) axis, promotes vascular smooth muscle cell pyroptosis and calcification via the Ca^2+^-CaMKII-NOD-like receptor family pyrin domain-containing 3 (NLRP3) pathway. In addition, Piezo1 contributes to inflammatory responses through the Piezo1-Src-signal transducer and activator of transcription 3 (STAT3) axis. Furthermore, low shear stress-induced NETosis is mediated by the Piezo1-histone deacetylase 2 (HDAC2) axis, in which Piezo1 downregulation leads to HDAC2 upregulation. At the cellular level, Piezo1 participates in regulating endothelial mechanotransduction, smooth muscle cell phenotypic switching, and macrophage functional polarization and foam cell formation in a context-dependent manner, ultimately affecting plaque stability. Piezo1 activity is subject to positive regulation by mechanical stimuli, chemical agonists (Yoda1), and pathological factors [such as the G protein-coupled receptor 146 (GPR146)-cAMP-CREB1 axis], as well as negative regulation by GsMTx4 and various natural compounds [salvianolic acid B, hydroxysafflor yellow A (HysA), and kaempferol]. This review systematically delineates the multicellular roles of Piezo1 throughout the full course of atherosclerosis and its integration with inflammatory and metabolic responses, providing a theoretical foundation for targeted intervention strategies. Future research should focus on the development of highly selective modulators, optimization of cell-specific delivery systems, and systematic integration of mechanotransduction with inflammatory and metabolic pathways.

## 1 Introduction

Atherosclerosis is an inflammatory disease of large arteries and a leading cause of cardiovascular disease (CVD) (1). It is a chronic inflammatory vascular disease driven by both traditional and non-traditional risk factors (2). Piezo1 is a newly identified transmembrane mechanosensitive cation channel, initially recognized for its role in touch sensation, and has now been found to be widely expressed in the mammalian cardiovascular system (3), particularly in vascular smooth muscle cells (VSMCs) and endothelial cells. It exists as a homotrimeric transmembrane complex and regulates calcium influx. In addition to membrane stretch associated with blood pressure, local blood flow-induced shear stress also serves as a key activator of this mechanosensitive channel (4). Shear stress has been demonstrated to regulate Piezo1 expression in endothelial cells, with the most pronounced changes observed at an intensity of 4.14 dyne/cm^2^, suggesting that Piezo1 may participate in the development of atherosclerosis induced by low shear stress (5). In recent years, accumulating evidence has highlighted the critical roles of mechanosensitive channels in myocytes, cardiac fibroblasts, vascular tone regulation, atherosclerosis, hypertension, nitric oxide (NO) production, and baroreceptor reflexes, underscoring the therapeutic potential of targeting mechanosensitive channel modulators in CVD (4). The cardiovascular system delivers blood throughout the body, thereby continuously imposing mechanical forces on cells and tissues. Consequently, it is enriched with mechanosensory structures capable of responding to and adapting to changes in mechanical stimuli. Dysregulation of cardiac function mediated by mechanosensitive channels may contribute to pathological conditions, including atherosclerosis and cardiac hypertrophy (6). Notably, Piezo1 plays a dual role in maintaining cardiovascular health: on one hand, its activity is essential for physiological cardiovascular function; on the other hand, its overexpression under extreme mechanical stimulation may promote adverse outcomes such as inflammation (7). This review summarizes recent advances and outstanding challenges in understanding the role of Piezo1 in cardiovascular health and presents novel perspectives for exploiting Piezo1 as a potential therapeutic target for atherosclerosis. Although several reviews have summarized the role of Piezo1 in CVD and atherosclerosis, the present review distinguishes itself from previous works in several important aspects. First, while earlier reviews predominantly focused on endothelial cells, we provide a comprehensive analysis of Piezo1 function across four major vascular cell types—endothelial cells, smooth muscle cells, macrophages, and neutrophils—covering the entire temporal continuum of atherosclerosis from initiation to plaque destabilization. Second, we integrate mechanotransduction with inflammatory and metabolic responses across multiple vascular cell types, moving beyond the classical single-pathway view that has dominated prior Piezo1 reviews. Third, we incorporate recent advances published within the last two years, including the Piezo1/BHLHE40/SLC7A11 axis in endothelial ferroptosis suppression (8, 9), the GPR146-cAMP-CREB1-Piezo1 regulatory module (10), the Talin1-Piezo1-YAP signaling node (11), and the Piezo1-SIRT4 endothelial protective axis (12)—mechanisms that were unavailable at the time of earlier reviews. Fourth, we provide a critical appraisal of Piezo1-targeted therapeutic strategies, encompassing both natural-product-based inhibitors (salvianolic acid B, HysA, and kaempferol) (13–15) and nanotechnology-enabled targeted delivery systems (16), with a focus on their translational potential and current limitations. Collectively, this review offers an updated, integrated, and clinically oriented perspective on Piezo1 in atherosclerosis that extends substantially beyond the scope of previous publications. In the following sections, we systematically discuss the biological functions, signaling pathways, cell-type-specific roles, regulatory mechanisms, and therapeutic implications of Piezo1 in atherosclerosis.

## 2 Biological functions and signaling pathways of Piezo1

### 2.1 Biological functions of Piezo1

As a mechanosensitive ion channel, Piezo1 is widely expressed in the cardiovascular system and participates in the regulation of various physiological and pathological processes. Studies have demonstrated that Piezo1 mediates adaptive changes in vascular structure and function by sensing hemodynamic shear forces, such as laminar shear stress (LSS) and OSS, thereby playing a critical role in vascular remodeling (VR) (17). Furthermore, Piezo1 is involved in blood pressure regulation; its deficiency impairs endothelium-dependent NO release, leading to hypertension (18). In the context of inflammation, Piezo1 channel activation promotes the expression of endothelial inflammatory mediators such as nuclear factor kappa-B (NF-*κ*B), tumor necrosis factor-alpha (TNF-α), and vascular cell adhesion molecule-1 (VCAM-1), thereby accelerating the progression of atherosclerotic plaques (19, 20). Notably, low shear stress-induced Piezo1 downregulation can also promote NETosis through HDAC2 upregulation (19). Moreover, Piezo1 influences autophagy and metabolic reprogramming through the Piezo1/YAP signaling pathway, for instance, by suppressing microtubule-associated protein 1A/1B-light chain 3-II (LC3-II) expression and promoting p62 accumulation, ultimately resulting in endothelial dysfunction (21).

### 2.2 Molecular mechanisms and signaling pathways of Piezo1

#### 2.2.1 Calcium-dependent signaling pathways

The activation of Piezo1 is strictly dependent on calcium influx, which in turn triggers multi-layered signaling cascades. Under OSS conditions, Piezo1-mediated calcium influx activates CaM/calcium/CaMKII, subsequently promoting the phosphorylation of FAK and Src, ultimately driving YAP nuclear translocation and inducing endothelial inflammatory responses (20). This Piezo1-Ca^2+^/CaM/CaMKII-FAK/Src-YAP axis has been identified as a key mechanotransduction pathway for OSS-induced endothelial inflammation (20). Additionally, this Piezo1-CaMKII axis suppresses the antioxidant function of nuclear factor erythroid 2-related factor 2, thereby exacerbating oxidative stress injury (22). In VSMCs, Piezo1 serves as an important regulator of vascular calcification through Ca^2+^-CaMKII-mediated NLRP3 inflammasome activation and subsequent pyroptosis; its downstream effector CaMKII not only initiates NLRP3 inflammasome assembly and pyroptosis but also interacts with Runt-related transcription factor 2 (RUNX2) to promote the expression of osteogenic proteins (23). In cardiomyocytes, Piezo1-mediated calcium overload activates calcineurin and calpain, promoting pathological cardiac hypertrophy (24). Together, these findings indicate that Ca^2+^-CaMKII signaling is a shared node in multiple vascular and cardiac cell types, but its downstream consequences are highly context-dependent, ranging from endothelial inflammation and VSMC calcification to cardiomyocyte hypertrophy. This cell-type specificity should be considered when targeting CaMKII-related pathways.

Beyond the aforementioned effects, calcium signaling also exerts sustained functions through transcriptional regulatory mechanisms. Studies have identified the Piezo1/Ca^2+^/calcineurin/nuclear factor of activated T cells 2 (NFAT2)/histone deacetylase 1 (HDAC1)/basic helix-loop-helix family member e40 (BHLHE40)/solute carrier family 7 member 11 (SLC7A11) axis as another dominant mechanotransduction pathway (8, 9). Upon shear stress-induced Piezo1 activation and calcium influx, calcineurin induces NFAT2 nuclear translocation, and NFAT2 recruits HDAC1 to form a transcriptional complex. This complex directly drives BHLHE40 expression, which in turn binds to the SLC7A11 promoter to upregulate this cysteine transporter, thereby suppressing endothelial ferroptosis and maintaining vascular homeostasis (8, 9).

Furthermore, exercise exerts cardiovascular protective effects through the activation of Piezo1 and transient receptor potential (TRP) channels, among which TRP channels participate in this protective mechanism by reducing transient receptor potential canonical 3 (TRPC3)-Nox2 complex formation and mediating transient receptor potential vanilloid 4 (TRPV4)-induced Ca^2+^ influx (25).

#### 2.2.2 Piezo1/YAP signaling pathway

The interplay between Piezo1 and the HIPPO-YAP signaling pathway plays an important role in VR and atherosclerosis. Disturbed flow or oxidized low-density lipoprotein (Ox-LDL) has been shown to upregulate Piezo1 expression, enhance YAP protein expression and nuclear translocation, and inhibit endothelial cell autophagy, thereby exacerbating vascular dysfunction (21). In addition, Piezo1 cooperates with the myokine irisin to regulate YAP activity, influencing the biomechanical properties of cardiovascular tissues (25). Talin1, a membrane-associated cytoskeletal protein widely expressed in mammals, has recently been identified as an important regulatory node of the Piezo1-YAP axis. Under inflammatory stimulation, TNF-α and OSS in endothelial cells (ECs) activate Piezo1-mediated Ca^2+^ influx, which in turn activates Talin1 to regulate YAP expression, ultimately promoting endothelial inflammation (11). Notably, Talin1 expression is elevated in the serum of patients with coronary artery disease and in ECs of atherosclerotic plaques in apolipoprotein E-knockout (ApoE-KO) mice, suggesting that it may serve as an important regulator of endothelial inflammation and a potential anti-inflammatory therapeutic target for atherosclerosis (11). Although YAP activation is often downstream of Ca^2+^-CaMKII signaling, recent evidence places Talin1 as an additional regulatory node that may integrate mechanical and inflammatory inputs. However, whether Talin1-Piezo1-YAP signaling is endothelial-specific or shared across vascular cells remains unresolved.

#### 2.2.3 Inflammatory and immunoregulatory pathways

Piezo1 promotes vascular inflammation through multiple mechanisms. In atherosclerosis, the Piezo1-Src-STAT3 axis has been shown to enhance endothelial inflammatory factor expression and accelerate plaque formation (26). Moreover, under LSS conditions, downregulation of Piezo1 leads to HDAC2 upregulation, which promotes neutrophil NETosis and may contribute to a pro-thrombotic state; however, plaque instability was not directly assessed (19). In macrophages, Piezo1 activation enhances inflammatory responses through calcium-dependent calpain activation, leading to renal fibrosis and epithelial-mesenchymal transition (27). Although neutrophil extracellular traps (NETs) capture and neutralize pathogens during host defense, excessive or dysregulated NET formation (termed NETosis) may exacerbate inflammation and contribute to thrombotic complications such as atherosclerosis and valvular lesions (28). OSS promotes Src phosphorylation at Y416 in endothelial cells, and Piezo1 is essential for this regulatory process. Overexpression of constitutively active Src promotes endothelial inflammation and phosphorylation of STAT3 at Y705 and its nuclear translocation. Disturbed flow promotes endothelial inflammation and atherogenesis through the Piezo1-Src-STAT3 pathway (26). Among these inflammatory mechanisms, the Piezo1-Src-STAT3 axis and Piezo1-HDAC2-NETosis axis are supported mainly by endothelial and neutrophil studies, whereas the macrophage calpain pathway has been characterized largely in non-atherosclerotic disease models. This evidence imbalance should be considered when extrapolating to human atherosclerosis.

#### 2.2.4 Metabolic reprogramming and its regulatory role

Piezo1-mediated mechanical stimuli trigger metabolic reprogramming of VSMCs, promoting foam cell formation. This process involves the phospholipid/arachidonic acid signaling pathway and is associated with epigenomic remodeling (29). In addition, downregulation of Piezo1 can lead to histone deacetylase 2 (HDAC2)-dependent NETosis (19), suggesting a potential intersection between Piezo1 signaling and epigenetic regulatory machinery. However, the mechanistic links between Piezo1 and epigenetic modifications remain largely unexplored. Thus, while metabolic reprogramming is an emerging theme in Piezo1 biology, current evidence is insufficient to establish a direct causal role for Piezo1 in epigenetic regulation during atherosclerosis.

## 3 Functions of Piezo1 in different cell types and its role in atherosclerosis

### 3.1 Function of Piezo1 in ECs

Piezo1 plays an important role as a mechanosensor in ECs. As a mechanically sensitive ion channel with a transmembrane trimeric structure, Piezo1 precisely detects mechanical stimuli such as blood flow-induced shear stress and participates in various physiological processes through calcium-dependent mechanotransduction (Section 2.2.1) (30, 31). Studies have shown that Piezo1 serves as a key mediator of vascular responses during exercise, triggering communication between ECs and VSMCs through membrane depolarization, thereby precisely regulating vasoconstrictive responses (32). During cerebrovascular development, Piezo1 promotes pericyte proliferation through activation of the Notch signaling pathway, expanding our understanding of how hemodynamic forces regulate vascular development (33). Recent studies have revealed a novel mechanism of the Piezo1-SIRT4 axis in endothelial protection: Piezo1 channel activity suppresses LOX-1-mediated Ox-LDL uptake by regulating SIRT4 expression, and this axis may modulate endothelial cell responses to shear stress, providing a protective mechanism against Ox-LDL-induced endothelial dysfunction and pathological injury (12). Endothelial Piezo1 is the most extensively characterized among vascular cell types, with consistent pro-inflammatory and pro-atherogenic effects in both *in vitro* and *in vivo* models. However, most mechanistic studies rely on HUVECs or mouse models, and whether these findings translate to human arterial endothelium remains to be established.

### 3.2 Function of Piezo1 in smooth muscle cells

In VSMCs, Piezo1 functions as an important mechanotransducer linking extracellular matrix stiffness to phenotypic modulation. Piezo1 activation promotes VSMC proliferation and migration through the Ca^2+^-CaMKII-calcineurin-YAP/TAZ axis described in Section 2.2.1, whereas VSMC-specific Piezo1 deletion inhibits disturbed flow-induced proliferation, migration, and neointimal hyperplasia *in vivo* (34). Consistent with this, mechanical stimulation alone is sufficient to induce the switch from a contractile to a synthetic phenotype, a process dependent on Piezo1 and associated with increased proliferative and migratory capacity (34). In addition, Piezo1 mediates VSMC calcification via Ca^2+^-CaMKII-NLRP3 signaling (as discussed in Section 2.2.1) and through stiffness-induced BMP2/RUNX2 activation, which is attenuated by GsMTx4 or Piezo1 knockdown, with reduced calcium deposition and restored SM22*α* expression (35). Piezo1 also regulates VSMC volume and hypertrophic responses to matrix rigidity by promoting calcium influx, PKC activation, and aquaporin-1 expression, with both Piezo1 and aquaporin-1 upregulated during VSMC phenotypic modulation in atherosclerosis (36). Finally, Piezo1 activation drives fibrotic gene expression, including type I/III collagens and *α*-SMA, via the Hippo pathway, potentially contributing to fibrous cap formation (37). Collectively, these findings indicate that Piezo1 in VSMCs is not merely a marker of plaque instability but a multifunctional regulator of proliferation, migration, phenotypic switching, calcification, hypertrophy, and matrix remodeling. Nevertheless, most evidence comes from injury or calcification models rather than authentic atherosclerotic plaques, and the relative contribution of each function to plaque progression remains to be defined.

### 3.3 Function of Piezo1 in macrophages

Piezo1 in macrophages serves as an important mechanosensor capable of precisely detecting changes in extracellular matrix stiffness. Studies have demonstrated that Piezo1-deficient macrophages exhibit attenuated inflammatory responses and enhanced wound healing capacity (38). Further investigations have revealed that Piezo1 forms a positive feedback regulatory loop with the actin cytoskeleton, a mechanism that may be critical for controlling macrophage polarization (38). Notably, the role of Piezo1 in macrophages appears to be context-dependent, although the direction of the net effect remains unresolved. Myeloid-specific Piezo1 deletion reduces plaque formation in Ldlr^⁻/⁻^ mice, indicating a pro-atherogenic role under genetic manipulation (39). In contrast, acute pharmacological activation with Yoda1 enhances macrophage phagocytosis and promotes foam cell apoptosis, suggesting a potentially protective function (40). Several factors may explain these seemingly opposing findings. First, genetic deletion eliminates Piezo1 throughout macrophage development, potentially inducing compensatory changes in lipid metabolism and inflammatory baseline, whereas pharmacological activation targets mature macrophages acutely. Second, the lipid microenvironment directly modulates channel behavior: Ox-LDL and 7-ketocholesterol potentiate Piezo1 currents and slow inactivation, whereas DHA inhibits Piezo1 activity (41). This indicates that local lipid composition can shape Piezo1 output, but it does not by itself explain the genetic-versus-pharmacological discrepancy. Third, Piezo1 activation promotes M1 polarization on stiff matrices but may support M2-mediated repair under distinct biomechanical conditions (38). Fourth, gain-of-function and genetic loss-of-function studies both point to a pro-atherogenic role (39) and therefore do not explain the protective effects reported with Yoda1. Collectively, the apparent contradiction may reflect differences in experimental manipulation (developmental genetic deletion versus acute pharmacological activation), lipid environment, and disease stage; however, current evidence is insufficient to determine whether a true functional dichotomy exists. These context-dependent effects are summarized in Table 1, which highlights the need to distinguish genetic deletion from pharmacological activation when interpreting Piezo1 function in macrophages.

**Table 1:** Summary of evidence for Piezo1 manipulation across vascular cell types in atherosclerosis.

Cell type	Piezo1 manipulation	Major phenotype	Effect on atherosclerosis	Level of evidence	References
Endothelial cells/HUVECs, ApoE^⁻/⁻^ mice	Endothelial-specific Piezo1 deletion	↓ YAP/NF-*κ*B, ↓ inflammation, ↑ eNOS	Reduced plaque formation	Strong (genetic + *in vivo*)	(20)
Endothelial cells/HUVECs	Yoda1 (acute)	↑ Ca^2+^, ↑ YAP nuclear translocation	Endothelial dysfunction	Preliminary (pharmacological *in vitro*)	(44)
VSMCs/Mouse carotid ligation	VSMC-specific Piezo1 knockout	↓ proliferation, ↓ migration, ↓ neointimal hyperplasia	Reduced neointimal hyperplasia; indirect for atherosclerosis	Strong for neointima; Indirect for atherosclerosis	(34)
VSMCs/*in vitro*	Piezo1 knockdown	↓ stiffness-induced calcification, ↓ BMP2/RUNX2	Reduced vascular calcification; indirect for atherosclerosis	Moderate (genetic *in vitro*; indirect for atherosclerosis)	(35)
VSMCs/*in vitro*	GsMTx4	↓ stiffness-induced calcification, ↓ BMP2/RUNX2	Reduced vascular calcification; indirect for atherosclerosis	Preliminary (non-selective, *in vitro*; indirect for atherosclerosis)	(35)
Macrophages/ApoE^⁻/⁻^ mice	Yoda1 (acute)	↑ phagocytosis, ↑ foam cell apoptosis	Potential plaque stabilization; context-dependent and not definitive	Moderate (pharmacological + *in vivo*)	(40)
Macrophages/*in vitro*	Gain-of-function mutation	↑ Ox-LDL uptake, ↑ foam cell formation	Pro-atherogenic mechanism; *in vivo* not assessed	Moderate (genetic *in vitro*)	(39)
Macrophages/Ldlr^⁻/⁻^ mice	Myeloid-specific Piezo1 deletion/knockout	↓ Ox-LDL uptake, ↓ plaque development	Reduced atherogenesis	Strong (genetic + *in vivo*)	(39)
Neutrophils/*in vitro*, *in vivo*	Piezo1 downregulation	↑ NETosis, ↑ pro-thrombotic scaffold	Potential pro-thrombotic effect; plaque instability not directly assessed	Moderate for NETosis; Indirect for plaque instability	(19)
Macrophages/*in vitro*	Salvianolic acid B (Piezo1 inhibitor)	↓ Ca^2+^ influx, ↓ foam cell formation	Anti-atherosclerotic	Preliminary (pharmacological *in vitro*; Piezo1 dependency not established)	(15)
Macrophages/*in vitro*	HysA	↓ Piezo1-YAP/JNK, ↓ foam cell formation	Potential anti-atherosclerotic effect	Preliminary (molecular docking evidence only)	(14)
Macrophages/*in vitro*	Kaempferol	↓ Piezo1-mediated Ca^2^⁺ influx; modulates CD36-dependent ROS and MAPK/NF-κB/Nrf2/HO-1 signaling	Potential anti-atherosclerotic effect	Moderate (pharmacological *in vitro*; Piezo1 dependency validated by Piezo1^*Δ*LysM knockout)	(13)

Evidence grading: **Strong:** cell-type-specific genetic manipulation with an *in vivo* atherosclerosis endpoint and direct target validation.

**Moderate:** (i) genetic manipulation *in vitro* with direct target validation; (ii) pharmacological manipulation *in vitro* with genetic target validation; or (iii) pharmacological manipulation *in vivo*/*ex vivo* with target validation and an atherosclerosis endpoint.

**Preliminary:** *in vitro* pharmacological data, non-selective inhibitor data, or lack of direct target engagement.

**Indirect:** the model is not atherosclerosis (e.g., carotid ligation-induced neointima or vascular calcification) and is therefore not direct evidence for atherosclerosis.

GsMTx4 is not selective for Piezo1; it also inhibits Piezo2 and several TRP channels. Therefore, GsMTx4-only evidence is graded no higher than Preliminary, and Piezo1-specific conclusions require genetic or siRNA-mediated validation.

HUVECs, human umbilical vein endothelial cells; VSMCs, vascular smooth muscle cells; Ox-LDL, oxidized low-density lipoprotein; NETosis, neutrophil extracellular trap formation; KO, knockout.

### 3.4 Function of Piezo1 in neutrophils

In neutrophils, low shear stress promotes NETosis through downregulation of Piezo1. As introduced in Section 2.2.3, this downregulation leads to decreased intracellular Ca^2^⁺ levels and increased HDAC2 expression, which in turn elevates ROS production and induces NETosis (19). This process not only modulates local inflammatory responses but also provides a scaffold for thrombus formation through the production of NETs, potentially contributing to the development of atherosclerotic complications. This finding offers a new perspective on the mechanisms of thrombosis in atherosclerosis.

### 3.5 Function of Piezo1 in other cell types

Beyond the aforementioned cell types, Piezo1 also plays an important role in cardiomyocytes. As introduced in Section 2.2.1, mechanically activated Piezo1 channels transduce mechanical stretch in cardiomyocytes into calcium and reactive oxygen species signals, a process essential for maintaining normal cardiac mechanical activity (42). Aberrant expression or function of Piezo1 may lead to dysregulation of calcium and reactive oxygen species signaling, thereby contributing to cardiomyopathy. These findings broaden our understanding of the role of Piezo1 in the cardiovascular system.

Across cell types, a consistent theme emerges: Piezo1 is not uniformly pro-atherogenic. Genetic deletion and pharmacological activation often yield divergent outcomes, particularly in macrophages, whereas endothelial and VSMC evidence is more congruent. Neutrophil data remain largely descriptive. This cell-type-specific heterogeneity implies that therapeutic targeting of Piezo1 must consider not only the cell type but also the mode of intervention and disease stage. The cell-type-specific functions of Piezo1 are summarized in Figure 1.

**Figure 1:**
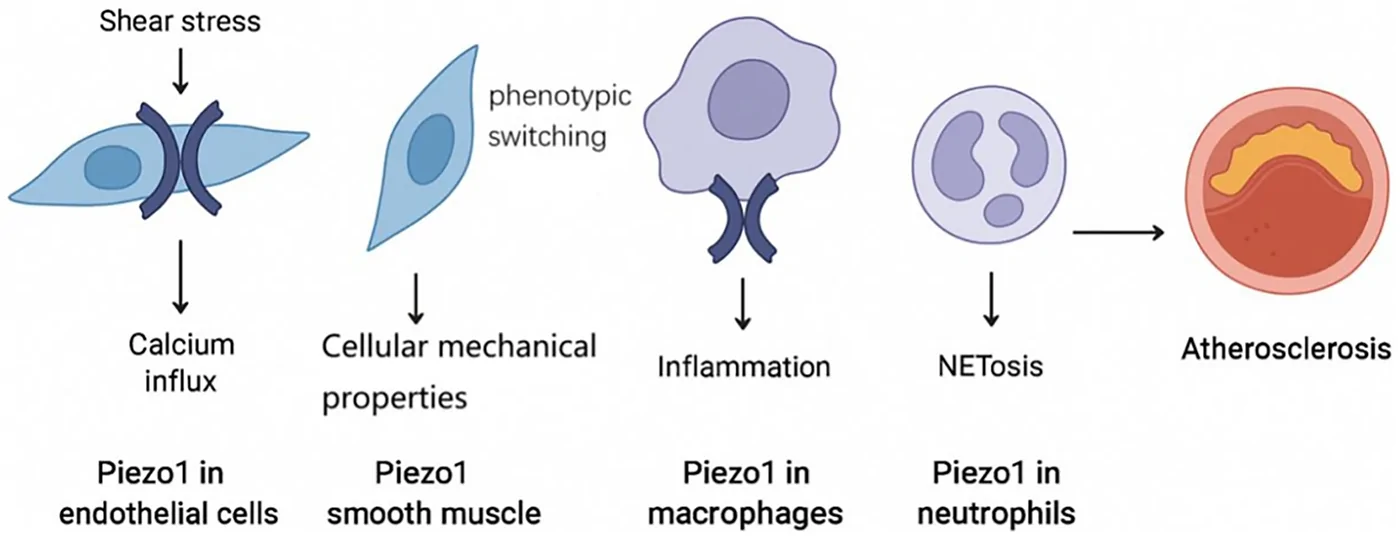
**Functions of Piezo1 in different vascular cell types and its role in atherosclerosis.** Piezo1 is expressed in endothelial cells, smooth muscle cells, macrophages, and neutrophils, where it mediates distinct mechanotransduction events that collectively contribute to atherosclerosis. In endothelial cells, Piezo1 activation triggers calcium influx. In smooth muscle cells, it regulates cellular mechanical properties and phenotypic switching. In macrophages, it regulates inflammation and macrophage polarization. In neutrophils, it induces NETosis (neutrophil extracellular trap formation). These cell-type-specific actions converge to drive the initiation and progression of atherosclerosis.

## 4 Regulatory mechanisms of Piezo1 and their roles in atherosclerosis

### 4.1 Positive regulatory mechanisms of Piezo1

#### 4.1.1 Regulation by mechanical stimuli

As a mechanosensitive ion channel, the activity of Piezo1 is finely regulated by various mechanical stimuli. Recent studies have demonstrated that different types of fluid shear stress exert distinct effects on Piezo1 activation. OSS specifically promotes Piezo1-dependent phosphorylation of Src kinase at the Y416 site in ECs, a process that plays an essential role in inflammatory responses in atherosclerosis-prone vascular regions (26). Notably, a clear dose-response relationship exists between shear stress intensity and Piezo1 activation, providing important clues to the molecular mechanisms by which low shear stress promotes atherosclerosis (5). Under physiological conditions, LSS triggers Piezo1-mediated mechanotransduction in ECs, leading to adrenomedullin release. This in turn activates the Gs protein-coupled cAMP signaling pathway, thereby regulating basal NO synthesis and maintaining vascular tone and blood pressure homeostasis (43). This precise mechano-chemical signal conversion constitutes the molecular basis for the rapid vascular response to hemodynamic changes.

#### 4.1.2 Activation by chemical agonists

Yoda1 is currently a widely used selective small-molecule agonist of Piezo1 and exhibits multiple regulatory effects in experimental studies. Yoda1 treatment significantly increases intracellular calcium levels in ECs and promotes nuclear translocation of the transcriptional coactivator YAP, a process closely associated with endothelial dysfunction (44). In macrophages, Yoda1 exerts context-dependent effects. In *in vitro* foam cell formation assays, Yoda1 promotes macrophage-derived foam cell formation, which is reversed by salvianolic acid B (15). In contrast, in ApoE^−/−^ mice and in independent *in vitro* functional assays, Yoda1 enhances macrophage phagocytosis and promotes foam cell apoptosis (40). Mechanistically, Yoda1-induced Piezo1 activation triggers Ca^2^⁺ influx, F-actin rearrangement, mitochondrial fragmentation, and increased mitochondrial ROS, accompanied by increased expression of anti-inflammatory genes (40). Inhibition of macrophage Piezo1 leads to lipid accumulation and foam cell formation, whereas Piezo1 activation promotes foam cell apoptosis (40). These findings suggest that the net effect of Yoda1 on macrophage lipid handling depends on the experimental model, lipid environment, and endpoint measured, rather than being uniformly pro- or anti-atherogenic.

#### 4.1.3 Upregulation under pathological conditions

During the development and progression of atherosclerosis, multiple pathological factors collectively regulate Piezo1 expression and activity. Studies have shown that Piezo1 exhibits distinct activation patterns under atheroprotective and pro-atherogenic shear stress, suggesting its dual role in vascular adaptive responses (45). Moreover, elevated levels of inflammatory cytokines and oxidative stress products in the inflammatory microenvironment significantly enhance Piezo1 activity, exacerbating endothelial dysfunction and vascular inflammation (44). Recent studies have further revealed a mechanism of GPR146 as an upstream regulator of Piezo1: GPR146 transcriptionally upregulates Piezo1 expression by enhancing CREB1 binding to the Piezo1 promoter region; GPR146 couples to G*α*s and activates the cAMP-CREB1-Piezo1 signaling pathway, participating in the regulation of hypertension and VR (10). These findings provide a molecular basis for the interplay between mechanical forces and biochemical signals in atherosclerosis.

### 4.2 Negative regulatory mechanisms of Piezo1

#### 4.2.1 Regulation by inhibitors

GsMTx4 is a widely used mechanosensitive channel inhibitor that blocks Piezo1 but is not selective for Piezo1; it also inhibits Piezo2 and several TRP channels. Therefore, GsMTx4 data should be interpreted as supporting, rather than proving, a role for Piezo1, and should be confirmed by genetic or siRNA-mediated Piezo1 manipulation. In atherosclerosis research, GsMTx4 treatment not only retards plaque progression but also effectively alleviates disturbed flow-induced endothelial inflammation (20). At the molecular level, GsMTx4 significantly reduces the expression of key inflammatory mediators within atherosclerotic plaques, including c-Jun N-terminal kinase (JNK), TNF-α, NF-*κ*B, and VCAM-1 (44). In colitis models, GsMTx4 inhibits Piezo1 channel activity, downregulates pro-inflammatory mediators such as Il1b, Il6, and Ptgs2 in colonic tissues, and selectively suppresses IL-6 production in macrophages and dendritic cells without affecting Th cell balance (46). These findings support a potential role for Piezo1 in inflammatory diseases, but GsMTx4-based conclusions require confirmation with Piezo1-specific genetic or pharmacological tools.

Salvianolic acid B is a natural bioactive compound that has been identified as a multi-target Piezo1 inhibitor. It dose-dependently inhibits Yoda1-induced calcium influx, blocks mechanically activated Piezo1 currents, and reverses Yoda1-promoted foam cell formation (15). Notably, in Piezo1 genetically depleted mice on a high-fat diet, salvianolic acid B showed no additional inhibitory effect on atherosclerotic plaques, a result the original study described as “insensitive to Piezo1 genetic depletion” (15). This does not constitute direct target validation, and the possibility that salvianolic acid B acts through additional Piezo1-independent mechanisms cannot be excluded.

#### 4.2.2 Regulation by genetic intervention

Genetic approaches have provided evidence for the functional role of Piezo1. Experiments have shown that Piezo1 gene deletion abolishes Yoda1-induced YAP nuclear translocation and calcium influx (44). At the cellular level, siRNA targeting Piezo1 attenuates Ox-LDL-induced endothelial inflammation and significantly reduces the expression of inflammatory mediators such as NF-*κ*B, JNK, and TNF-α (44). These genetic intervention results not only validate the critical role of Piezo1 in atherosclerosis but also identify potential targets for gene therapy.

#### 4.2.3 Physiological feedback regulation

Under physiological conditions, the body maintains a finely tuned Piezo1 negative feedback network. Piezo1 and Piezo2, as core mechanosensors in baroreceptors, trigger the baroreceptor reflex upon activation to rapidly regulate blood pressure. Selective knockout of these channel genes leads to increased blood pressure variability and persistent hypertension, closely resembling the clinical features of patients with baroreflex failure (47). Furthermore, physiological laminar flow promotes NO production through Piezo1, and NO may in turn suppress excessive Piezo1 activation via a cGMP-dependent pathway, forming a precise autoregulatory circuit (43).

Despite the availability of GsMTx4, Yoda1, and natural-product inhibitors, none has achieved cell-type selectivity or clinical validation. The same mechanical stimulus can be protective or detrimental depending on flow pattern and duration, and systemic Piezo1 inhibition carries potential risk given its physiological roles in baroreception and vascular tone. These limitations define the current translational gap.

## 5 Mechanisms by which Piezo1 promotes atherosclerosis

As summarized in Table 1, the strength of evidence for Piezo1 manipulation varies across cell types and experimental models, with genetic deletion in ApoE^⁻/⁻^ or Ldlr^⁻/⁻^ mice providing the strongest support for a pro-atherogenic role, while pharmacological activation with Yoda1 may exert context-dependent effects. The stage-specific mechanisms by which Piezo1 promotes atherosclerosis are illustrated in Figure 2.

**Figure 2:**
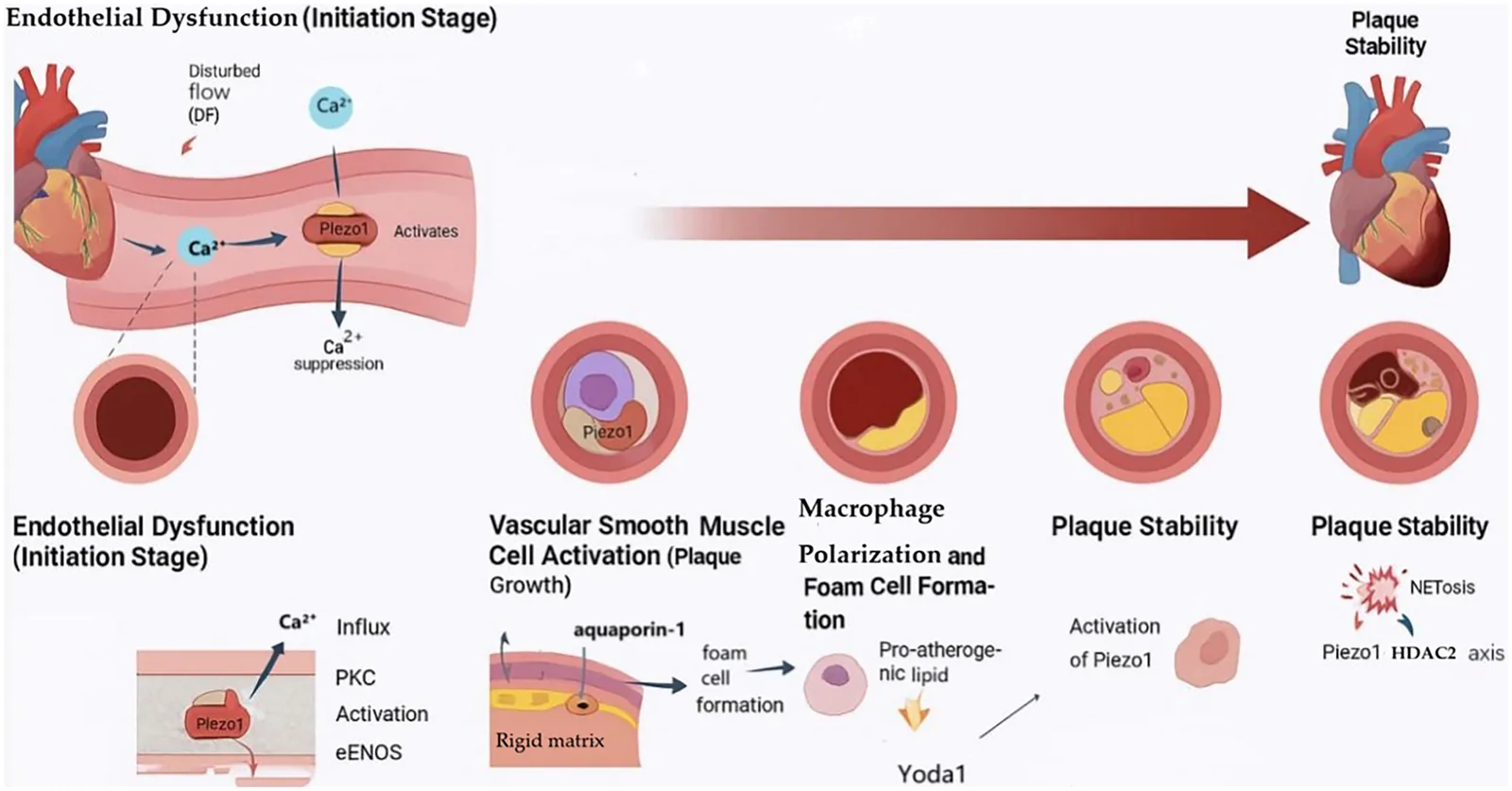
**Mechanisms by which Piezo1 contributes to atherosclerosis.** Piezo1 mediates stage-specific effects during atherosclerosis progression. In the initiation stage, disturbed flow (DF) activates endothelial Piezo1, leading to Ca^2+^ influx, PKC activation, and altered eNOS signaling, which contribute to endothelial dysfunction. During plaque growth, Piezo1 activation in vascular smooth muscle cells (VSMCs) promotes aquaporin-1 expression, foam cell formation, and a hypertrophic-like response on rigid matrix. In macrophages, pro-atherogenic lipids and Yoda1 activate Piezo1, driving foam cell formation and polarization. In advanced plaques, Piezo1 contributes to plaque instability through inflammation and apoptosis, while Piezo1 downregulation and HDAC2 upregulation mediate NETosis. The figure summarizes the multi-cellular and multi-stage roles of Piezo1 in atherosclerosis. DF, disturbed flow; PKC, protein kinase C; eNOS, endothelial nitric oxide synthase; VSMCs, vascular smooth muscle cells; NETosis, neutrophil extracellular trap formation.

### 5.1 Endothelial dysfunction (initiation stage)

At arterial bifurcations and curvatures, disturbed flow-induced OSS activates endothelial Piezo1 through the Ca^2+^/CaM/CaMKII-FAK/Src-YAP axis (Section 2.2.1), leading to YAP/NF-*κ*B-dependent endothelial inflammation (20). In addition, combined disturbed flow and Ox-LDL upregulate Piezo1 and impair autophagy (21). Endothelial-specific Piezo1 deletion attenuates high glucose-induced endothelial apoptosis and aortic inflammation while enhancing eNOS expression (22). These findings suggest that Piezo1 may serve as a molecular switch linking mechanical stimuli to endothelial inflammation, thereby initiating the early endothelial dysfunction that predisposes the vessel wall to plaque formation.

### 5.2 Vascular smooth muscle cell activation (plaque growth)

During the progression stage of atherosclerosis, phenotypic switching of VSMCs is an important driver of plaque growth. As detailed in Section 3.2, increased extracellular matrix stiffness activates Piezo1 channels in VSMCs, leading to PKC activation and aquaporin-1 upregulation, resulting in a hypertrophic-like response (36). Importantly, mechanical stimulation alone is sufficient to induce vascular smooth muscle cell switching to a synthetic phenotype, thereby promoting foam cell formation (29). In abdominal aortic aneurysm models, VSMCs enhance cytoskeletal stiffness through upregulation of *α*-actinin-2, providing the requisite mechanical force for Piezo1 activation and driving pathological VR (48). These observations highlight the central role of Piezo1 in vascular smooth muscle cell mechanosensation and phenotypic modulation, offering new insights into the mechanisms of plaque growth in atherosclerosis.

### 5.3 Macrophage polarization and foam cell formation

As detailed in Section 3.3, macrophage Piezo1 function is context-dependent and influenced by the lipid microenvironment and matrix stiffness (38, 41). Genetic and pharmacological manipulations produce divergent effects on foam cell formation and apoptosis (39, 40), which collectively contribute to plaque progression.

### 5.4 Plaque stability (advanced stage)

In advanced atherosclerosis, Piezo1 affects plaque stability through multiple mechanisms. Piezo1 activation increases intracellular calcium levels, thereby activating PKC and NF-*κ*B signaling pathways and promoting the release of inflammatory mediators (49). In the context of fibrous cap formation, this pathway may weaken stability by exacerbating inflammation, although whether Piezo1 directly regulates vascular smooth muscle cell-mediated collagen deposition remains to be determined. Vascular smooth muscle cell apoptosis is a major contributor to lipid core expansion and fibrous cap thinning, driven in part by Piezo1-mediated calcium overload and reactive oxygen species generation (29, 42). Furthermore, the Piezo1-HDAC2 axis mediates neutrophil NETosis under low shear stress, potentially contributing to a pro-thrombotic milieu; however, plaque stability was not directly evaluated (19).

Collectively, these stage-specific findings suggest that Piezo1 may act as a context-dependent switch rather than a simple driver of atherosclerosis. Early endothelial activation, VSMC phenotypic switching, macrophage polarization, and neutrophil NETosis are all modulated by Piezo1, but the net effect depends on cell type, lipid environment, and disease stage. This complexity explains why both Piezo1 inhibition and activation have been reported to be beneficial in different settings, and it underscores the need for stage-specific and cell-specific therapeutic strategies.

## 6 Strategies for targeting Piezo1 in the treatment of atherosclerosis progression

### 6.1 Therapeutic potential

Although these findings are primarily derived from preclinical models and have not yet been validated in human clinical trials, they provide mechanistic insights that may inform future therapeutic strategies. Given the important role of Piezo1 in CVD, targeting Piezo1 and its downstream signaling molecules may represent a potential therapeutic strategy. As discussed in Section 4.2, the non-selective Piezo1-blocking agent GsMTx4 attenuates atherosclerotic plaque formation and endothelial inflammation (20), but requires Piezo1-specific validation. Genetic or siRNA-mediated Piezo1 silencing suppresses YAP/TAZ activation, reduces inflammatory cytokine expression, and improves vascular function (44). Furthermore, targeting downstream effectors of Piezo1, such as HDAC2 and calpain, may offer new interventions for atherosclerosis, hypertension, and pulmonary hypertension (19, 27, 50).

### 6.2 Advances in drug development

As discussed in Sections 4.1.2 and 4.2.1, both Piezo1 agonists and inhibitors have been explored for atherosclerosis. Yoda1 exerts context-dependent effects on macrophages (Section 4.1.2) (15, 40); nevertheless, it exacerbates brain injury in intracerebral hemorrhage models (16), underscoring the need for more selective agonists. Among natural compounds, salvianolic acid B inhibits Piezo1 currents and reverses foam cell formation, although its protective effects on plaque formation were insensitive to Piezo1 genetic depletion (15); HysA alleviates atherosclerotic inflammation via Piezo1-YAP/JNK inhibition, although direct binding evidence is currently limited to molecular docking analysis (14). Kaempferol inhibits Piezo1-mediated Ca^2^⁺ influx and modulates CD36-dependent ROS and MAPK/NF-*κ*B/Nrf2/HO-1 signaling, with its Piezo1 dependency validated in macrophage-specific Piezo1 knockout mice (13). Direct binding evidence is still lacking for HysA and kaempferol. Current pharmacological strategies targeting Piezo1 are summarized in Figure 3. Overall, these strategies remain preclinical, and their cell-type specificity, long-term safety, and translational potential require further investigation.

**Figure 3:**
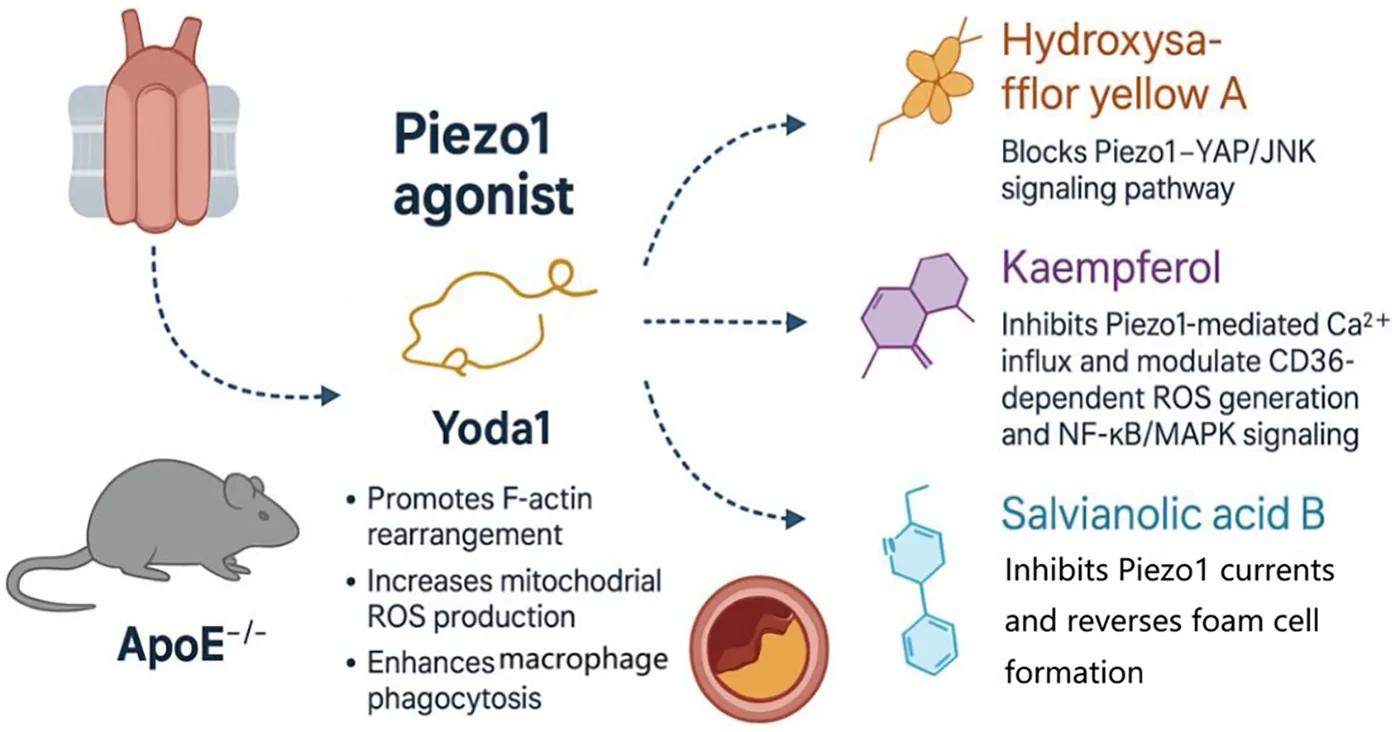
**Progress in drug development targeting Piezo1.** Yoda1, a selective Piezo1 agonist, promotes F-actin rearrangement, increases mitochondrial ROS (reactive oxygen species) production, and enhances macrophage phagocytosis in ApoE^⁻/⁻^ mice, leading to reduced plaque area and increased collagen content. Natural compounds including hydroxysafflor yellow A (blocks Piezo1–YAP/JNK signaling), kaempferol (inhibits Piezo1-mediated Ca^2+^ influx and modulates CD36-dependent ROS generation and NF-*κ*B/MAPK signaling), and salvianolic acid B (inhibits Piezo1 currents and reverses foam cell formation) have been identified as Piezo1 inhibitors with anti-atherosclerotic properties. These pharmacological strategies highlight the therapeutic potential of targeting Piezo1 in atherosclerosis. ROS, reactive oxygen species; ApoE^⁻/⁻^, apolipoprotein E-knockout; NF-*κ*B, nuclear factor kappa-B; MAPK, mitogen-activated protein kinase.

### 6.3 Exploration of novel therapeutic approaches

Nanotechnology offers new opportunities for Piezo1-targeted therapy, but current evidence remains preclinical. In intracerebral hemorrhage models, intracerebroventricularly administered GsMTx4, a non-selective mechanosensitive channel inhibitor with Piezo1-blocking activity, effectively reduces Piezo1 and aquaporin-4 (AQP4) expression, alleviates cerebral edema, and improves neurological outcomes (16). However, this study was not performed in atherosclerosis, and GsMTx4 is not selective for Piezo1. Whether intracerebroventricularly administered GsMTx4 can target plaque macrophages while avoiding systemic Piezo1 inhibition remains unknown. Therefore, this approach should be regarded as a preclinical hypothesis rather than a demonstrated therapeutic strategy for atherosclerosis.

Targeted intervention strategies based on the Piezo1 signaling network are emerging. For example, the traditional Chinese medicine compound GZTLT (Guizhitongluo Tablet) reduces plaque formation by inhibiting Piezo1 expression and calcium influx, thereby modulating NLRP3 inflammasome activation and pyroptosis (51); the combination of ginsenoside Rg1, notoginsenoside R1, and protocatechuic aldehyde improves endothelial function through the Piezo1-FAK-phosphoinositide 3-kinase/protein kinase B (PI3 K/Akt) pathway (52); and the anti-inflammatory effects of jatrorrhizine are partially dependent on Piezo1 regulation (53).

Among the natural compounds discussed, salvianolic acid B inhibits Piezo1 currents *in vitro*, although its *in vivo* protective effects were insensitive to Piezo1 genetic depletion (15); HysA currently lacks direct experimental binding evidence, whereas kaempferol has been validated using macrophage-specific Piezo1 knockout mice, although its binding evidence is currently limited to molecular docking (13, 14). This evidence hierarchy should guide future translation: compounds with validated Piezo1 dependency (e.g., kaempferol) should be prioritized for preclinical development, while others require further validation before therapeutic claims can be made.

## 7 Conclusions and future perspectives

This review systematically delineates the multicellular regulatory roles and molecular mechanisms of the mechanosensitive ion channel Piezo1 in the initiation and progression of atherosclerosis. Existing evidence demonstrates that Piezo1, by sensing changes in blood flow shear stress and extracellular matrix stiffness, transduces pathological mechanical stimuli into multi-layered cellular responses, including endothelial inflammation, vascular smooth muscle cell phenotypic switching, context-dependent macrophage polarization, and neutrophil NETosis, thereby participating in the full course of atherosclerosis from initiation to plaque destabilization. At the signaling pathway level, Piezo1 not only drives inflammation through the Ca^2+^/CaM/CaMKII-FAK/Src-YAP axis (20), but also suppresses endothelial ferroptosis and maintains vascular homeostasis through transcriptional regulatory mechanisms (such as the BHLHE40/SLC7A11 axis) (8, 9), and is subject to positive regulation by upstream signals such as GPR146-cAMP-CREB1 (10). These context-dependent outputs highlight the importance of distinguishing physiological versus pathological mechanosensing when considering Piezo1 as a therapeutic target. At the pharmacological level, multiple natural compounds (e.g., salvianolic acid B, HysA, and kaempferol) have been identified as Piezo1 modulators with anti-atherosclerotic potential (13–15), although their evidence levels vary, with salvianolic acid B shown to inhibit Piezo1 currents *in vitro* but its *in vivo* protective effects insensitive to Piezo1 genetic depletion, while direct binding evidence for HysA and kaempferol remains to be established, providing important candidate molecules for Piezo1-based intervention strategies.

(1) Spatiotemporal resolution of cell type-specific functions. Given the highly heterogeneous functions of Piezo1 across different vascular cell types, future studies should employ single-cell sequencing and lineage tracing technologies to precisely dissect the spatiotemporal dynamics of Piezo1 signaling pathways in various cell types and their relative contributions at different stages of atherosclerosis.
(2) Development of highly selective modulators. The selectivity and safety profiles of existing tool molecules (such as GsMTx4 and Yoda1) remain suboptimal. Future efforts should focus on rational drug design based on the three-dimensional structure of Piezo1 to develop small-molecule modulators with subtype selectivity and cell type-targeting properties.
(3) Mechanistic elucidation and clinical translation of natural products. Compounds such as salvianolic acid B, HysA, and kaempferol have demonstrated Piezo1-targeting potential (13–15); however, their direct binding sites, structure-activity relationships, and pharmacokinetic properties warrant systematic investigation.
(4) Systematic integration of mechanotransduction with inflammatory and metabolic pathways. How Piezo1 translates mechanical signals into metabolic reprogramming and inflammatory signaling is currently supported only by preliminary evidence (29). Future studies should integrate multi-omics approaches with biomechanical modeling to construct cross-scale regulatory networks.
(5) Clinical translational validation of targeted delivery systems. Nanotechnology-mediated Piezo1-targeted delivery has shown promise in animal models (16); however, its cell specificity, long-term safety, and scalable manufacturing processes remain to be addressed.
